# Sustainability of Nursing Leadership and Its Contributing Factors in a Developing Economy: A Study in Mongolia

**DOI:** 10.3389/fpubh.2022.900016

**Published:** 2022-05-25

**Authors:** Bing-Long Wang, Munkh-Ulzii Batmunkh, Oyunsuren Samdandash, Duumaam Divaakhuu, Wing-Keung Wong

**Affiliations:** ^1^School of Health Policy and Management, Chinese Academy of Medical Sciences and Peking Union Medical College, Beijing, China; ^2^Department of International Relations, National University of Mongolia, Ulaanbaatar, Mongolia; ^3^Department of Healthcare Administration, Asia University, Taichung City, Taiwan; ^4^Fintech & Blockchain Research Center, Department of Finance, Asia University, Taichung City, Taiwan; ^5^Big Data Research Center, Department of Finance, Asia University, Taichung City, Taiwan; ^6^Department of Medical Research, China Medical University, Taichung City, Taiwan; ^7^Department of Economics and Finance, The Hang Seng University of Hong Kong, Shatin, Hong Kong SAR, China

**Keywords:** nurse leadership, work environment, performance, problem-solving, transformational role

## Abstract

The sustainability of nursing leadership is a very important problem. Every country continually strives to find the best ways to advance in nurse management and patient care services. Nursing leadership is most desirable in the delivery of health care services. Since there is limited information about leadership skills in Mongolia, to solve the problem of the sustainability of nursing leadership, we carried out this study to explore factors contributing to the sustainability of nursing leadership and their correlation relatively to nurse managers in healthcare institutions. A sample of 205 nurse managers from all forms of health facilities participated in this study. The data were analyzed by descriptive, correlation, and multiple linear regression models using SPSS 19 version. The linear combination of the five independent variables was significantly related to the dependent variable (nurse leadership). Both the behavior and problem-solving are significant regressors of the dependent variable. The correlation analysis significance of the independent study variables, two were found to have a significant effect on nursing leadership: behavior and performance of nurses significantly and positively effect nursing leadership. The transformational role and nurse leadership produced a significantly positive Correlation coefficients give a direction of causation in the relationships of variables, and the multiple linear regression analysis says that two of the variables, namely, behavior and problem-solving, positively contribute to nursing leadership, two of the variables namely, work environment and performance nurse manager do not support; however, variable transformational ability majorly contributes to the sustainability of nursing leadership.

## Introduction

The sustainability of nursing leadership is a very important problem. Healthcare is one of the challenging industries that require complex demands, and needs successful recruitment strategies; however, it is quite difficult to select competent professionals and keep them for a longer period of time. As a growing segment of the population ages, each country strives to find the best way to improve its nursing management and patient care system. Nurses play an important role as doctors in the delivery of health care services. Due to the increased demand for nurse managers, the form of leadership is most desirable in the daily working environment of nurses (1–4).

Nurse managers engage in a range of leadership activities in their daily routine that some will naturally adopt an effective leadership style and provide higher leadership roles, while others may find the concept of leadership is difficult to understand or see themselves not so much competent. Nurse leaders should have rational thinking and exceptional communication skills that are measured by the positive influential ability to reach the goals of health care. The key role of nurse managers is to motivate their subordinates to be autonomous in making patient care decisions and perform safe patient care according to the standards of nursing practice (5–7).

Leadership is important in high-quality patient care and facilitating positive staff development in healthcare settings. Effective leadership significantly influences reducing turnover of nurses and increasing job satisfaction in the workplaces (8, 9). According to literature, leaders should be able to work under pressure and take immediate actions to solve problems, and, at the same time, be both taught and learned in the work environment. Nevertheless, leaders must show emotional intelligence to manage their own and others' feelings. In addition, leaders must have a transformational role to influence their own and others' performances that impact problem-solving in the workplace (9, 10).

Nowadays, the leadership role of nurses depends on rapid technological changes, communication style, information transparency, needs of patients, service quality, and compliance with regulations and standards (8). Besides, the nurse manager is a coach, while nurses provide high-quality patient service, stabilize workload and stress, and increase efficiency in the workplaces. Typically, the leadership of nurse managers is developed through specific educational activities by modeling and practicing competencies (11). Nevertheless, cultural differences influence nursing leadership, for instance, in Arabic countries, nurse managers have an integrative leadership role; in spite of it, in western countries, nurse managers prefer to be decentralized (12, 13).

With the notable shift in the healthcare needs of global populations, healthcare institutions across the world face enormous challenges to be more responsive and efficient, a responsibility that cannot be met without ensuring good quality of nursing care. Yet, due to inconsistent economic development, the quality of nursing varies significantly from country to country. In developing countries, such as Mongolia, nurses work, often under difficult circumstances, in health services that are grossly underfunded and are a vailable only to those who can pay (14).

Over the last decades, the health care industry in Mongolia has faced a series of problems, such as low quality in care provision, human resources scarcity, inadequate training, and insufficient ongoing education for nurses and nursing leadership, as well as poor working environments. In spite of that, nurses work hard to facilitate their resources to their job without considering the environment.

In brief, Mongolia is a landlocked developing country, which is between China and Russia, with a population of 3 million, the majority of which live in the capital city. As of 2020, the life expectancy in Mongolia was 69.9 years. By 2012, there were 9,916 registered nurses (see Table 1), while this number increased to 10,948 in 2016 (4, 15, 16).

**Table 1:** Number of hospital and nurses.

**Type of hospitals**	**Number of health care institution**	**Number of nurses**	**Nurse managers**
**Primary level health care institutions**			
1. Soum hospitals, including inter-soum hospitals in 21 aimags of Mongolia	271	1,165	87
2. Family hospital	221	785	41
Sub-Total	492	1,950	128
**Secondary level health care institutions**			
1. Provincial general hospital of 21 aimags	20	1,725	76
2. District hospitals in 9 districts of Ulaanbaatar	8	258	7
Sub-Total	28	1,983	83
**Tertiary level health care institutions**			
1. Specialized hospitals in UB	16	1,988	44
2. Regional diagnostic and treatment center	5	1,357	35
Sub-Total	21	3,345	79
**Other health care institutions**			
1. Private clinics	1,173	1,275	71
2. Hospitals of state agencies (Authority of border patrol, Railway organization)	45	406	24
3. Maternity hospitals	3	115	8
4. Other healthcare institutions	1,119	842	13
Sub-Total	2,340	2,638	116
Total	2,881	9,916	406

*The health care system in Mongolia is characterized by three levels: primary level health care institutions consist of family group infirmaries in Ulaanbaatar, the capital city, in aimag centers, and in soum and inter-soum hospitals in provinces. Secondary-level healthcare institutions include district general hospitals in Ulaanbaatar and provincial general hospitals. Tertiary-level hospitals are major specialized hospitals in Ulaanbaatar and 5 regional hospitals. Ministry of Health of Mongolia (17)*.

Studies discuss that insufficiency in autocratic nursing leadership is common within hospital settings of Mongolia, which is the main problem of this study (8). According to the literature, the common factors that have a positive effect on nursing leadership are work environment, performance, behavior, problem-solving, and transformational role (18), which are discussed in section Literature Review and Research Hypotheses more in detail. Thus, the purpose of this study is to explore factors that affect nursing leadership in healthcare institutions of Mongolia. We hope that this study will also serve as a catalyst for further exploration of influencing factors on leadership in developing countries. This study provides instruments in helping hospital administrators to meet the needs of long–term employment of nurses in their organizations. A greater understanding of nurse leadership changes people's minds and functions and increases healthcare quality and patient care services in hospitals of Mongolia. This study has a critical implication on Government policies and regulations on how to develop nurse managers in healthcare settings around the country.

The remainder of the paper is organized as follows. Section Literature Review and Research Hypotheses reviews relevant literature and describes the hypotheses to test. Section Methods presents the methodology. Empirical results are reported in section Data Analysis, while section Conclusions and Discussions presents the conclusions of the paper.

## Literature Review and Research Hypotheses

The theoretical foundation of this study is based on leadership theory, management theory, and psychological theory of nurse managers that influence the activities and competence of an individual or a group in efforts to have goals of achievement in a given situation. Leadership theory says that some people are born to be leaders, while, according to management theory, leadership is a position and a skill that can be earned and developed through years of experience (11, 19). According to the psychological theory, naturally, women have lower aggressiveness that restrains women from leadership positions. Nevertheless, gender plays an important role in the nursing profession and remained predominantly female (20, 21).

There are a number of definitions and typologies for the leadership role of nurse managers. The majority of studies used the theoretical framework of Hersey and Blanchard's Situational Leadership Model, Kouzes and Posner's Leadership Challenge, Burns' Transformational Leadership, Bass and Avolio's Transformational and Transactional Leadership, McLelland's Theory of Leadership Motivation (22). They found 20 factors that affect the leadership role and categorized the factors into four groups: [1] behaviors and practices; [2] traits and characteristics; [3] context and practice settings; and [4] educational activities.

Other scholars described nurse roles functions as an independent role function, a dependent role function, and an interdependent role function (23), which are similar to the classification of managerial theory (18) as classified into three major roles: [1] interpersonal, derived from authority and status including the role's figurehead, leader, liaison; [2] informational, derived from interpersonal roles, including the role's monitor, disseminator, and spokesman, and [3] decisional, derived from a manager's information, including the roles of entrepreneurs, disturbance handlers, resource allocators, and negotiators.

As stated in the research of Ramey (5), the leadership role prevents turnover and promotes retention, which is economically important for hospitals and healthcare institutions. Koy et al. (9) found that nursing leadership plays an important role in nursing managers' job satisfaction, organizational commitment, and workplace empowerment.

**Figure 1:**
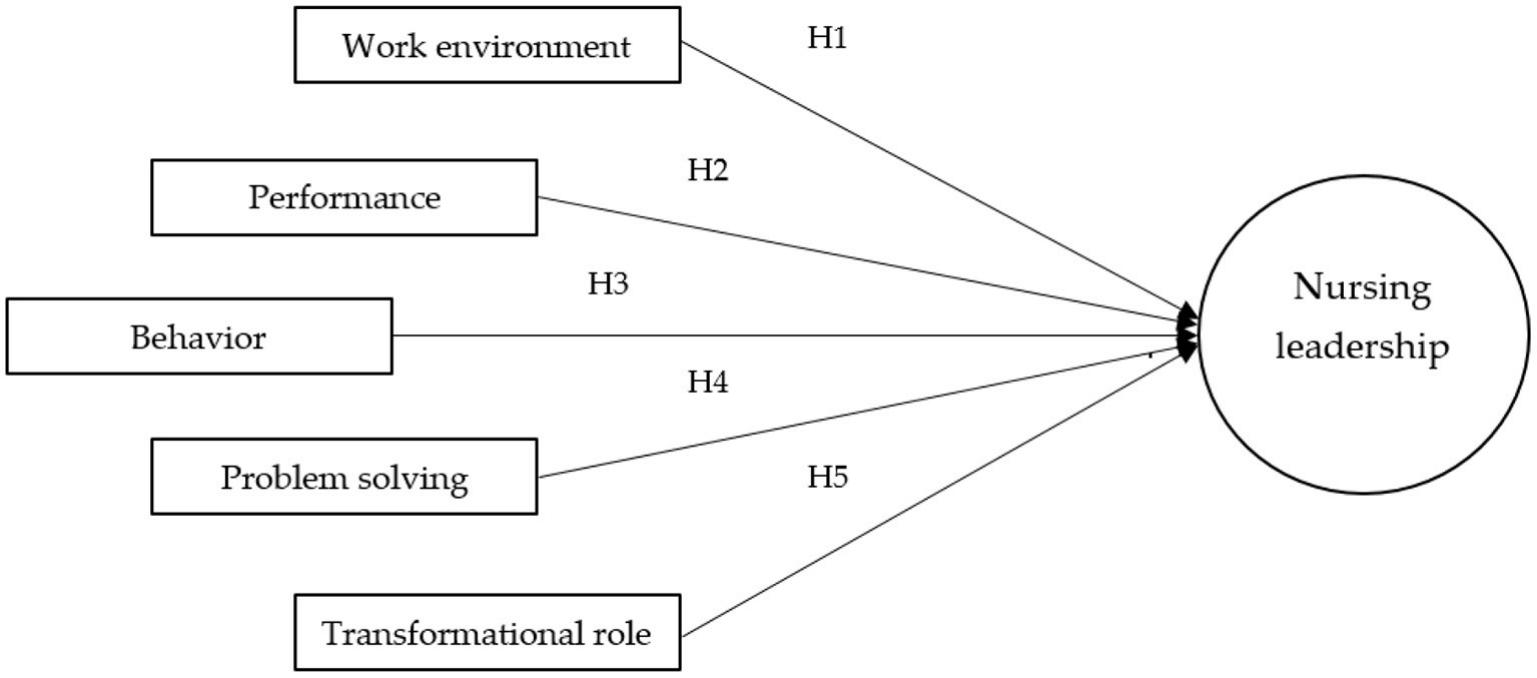
Theoretical framework.

Thus, this study makes a general proposition (see Figure 1) that factors, such as work environment, performance, behavior, problem-solving, and transformational role, affect positively nursing leadership.

### Work Environment

A nurse's role in the workplace encompasses illness prevention and care, health promotion and disabilities and palliative care, whereas a leader's role of nurses in the workplaces is to create a conducive work environment (23–25). Nurses are required to work overtime, and extra shifts are creating a stressful work environment. Therefore, nurse managers aim at maximizing nursing productivity and minimizing the direct and indirect costs of overtime work. Nursing has an important impact on hospital costs and the rational use of resources and reduced waste that reduce delivery of care cost and enable larger investment in quality (3, 11, 26).

Rajbhandary and Basu (3) identified that improving the work environment has to be identified as one retention strategy, so it is important to identify mechanisms to retain nurses and increase nurse satisfaction while improving the work environment and working conditions. In the healthcare system, a healthy work environment should be created for the appropriate nursing staff level. Nurse managers experience severe psychological stress and a heavy burden at work, which could have conflict in the work environment. A stressful work environment would likely constitute less autonomy, less control, and a lack of respect. Moreover, they create a safe environment for effective management of the conflict to stimulate personal growth and ensure quality patient care (9, 12, 20, 24).

Many researchers used the Revised Nursing Work Index (NWI –R) and Environment Scale of the Nursing Work index (PES-NWI) to measure factors in the work environment to support professional nursing practice, and explored that leader's role is a critical factor in the work environment (27). Clinical leaders foster a supportive work environment to empower their subordinate nurses in management positions (9). A positive leadership role encourages nurses in managerial positions to involve in a common organizational commitment that contributes to an optimal work environment (9, 28).

Casida (6) found that the leadership role of nurse managers is directly influenced by the nursing unit and organizational culture that is responsive to the external and internal perspectives forward to the hospital goals and vision. A positive work environment does not naturally occur, instead created and fostered by strong nurse leaders their visibility, accessibility, consultation, recognition, and support (27). Thus, the following hypothesis is set to test whether the work environment is positively related to nursing leadership:

**Hypothesis 1:** Work environment is positively related to nursing leadership.

### Performance

The performance of a nurse in a healthcare institution is an interaction between people to work together and help the patients, thereby reducing the power imbalance between the patient and the physician and creating dependency on the part of the patients. Nursing performance is critical to the management of a nursing ward and closely tied to role enhancement of nurse managers and job satisfaction (11, 26, 29).

Health care organizations, including nurse care departments face formidable challenges in improving nurse performance, which is the fundamental aspect to successfully excel in many organizational elements and effectively enhance health care quality to patients. Nurse managers with high performance successfully achieve their responsibility in an organization and have a positive influence on nursing leadership; however, nurse managers with weak performance spent considerable energy, articulating the importance of nursing to the organization (9, 21, 30).

**Hypothesis 2:** Performance is positively related to nursing leadership.

### Behavior

Koy et al. (9) state that demand for care is skyrocketing, and supply for a caregiver is plummeting that behavioral component is essential for nurse managers. Nursing intervention is defined as assisting a patient, significant others, and/or family to improve relationships by clarifying and supplementing specific role behaviors. Some researchers argue that a behavior element has a positive effect on the nurse manager's role based on the leader-member exchange theory. The behavior of nurse managers is most important in staff nurse satisfaction, engaging nurses in the work environment (27).

According to Nilsson et al. (25), role modeling of leadership behaviors by managers, clinical nurse specialists, and other colleagues is developed through a nurse leadership program. Theories of leadership also emphasize positive behaviors are the essential part for leaders. The development of leadership expertise has been described as a process of developing competencies and behaviors over time through education, preceptorship, and mentoring. Supportive interpersonal behavior at work is an important dimension of a nurse manager (11) that managerial support is directly impacted by the attitude and behaviors of the nurse leaders.

Several studies (29) used the Collaborative Behavior Scale created by Stichler (31) to determine the extent of collaboration behaviors that generally exist between nurses and nurse managers. Results of their study show that positive behavior influences positively the leadership role and favorable work environment. They conclude that bad behavior increases workload, turnover, lack of responsibility. Furthermore, the authors suggest that hospital management should stimulate the autonomy of the nurse managers by creating an environment in which career opportunities are clearly delineated in terms of behavior.

In reality, nurses exhibit diverse behaviors, and most of the nurses do not engage in effective conflict resolution, sharing ideas, understanding each other, and communication about what needs to be done for the patient. Therefore, we developed the third hypothesis to examine whether the behavior is positively related to nursing leadership:

**Hypothesis 3:** Behavior is positively related to nursing leadership.

### Problem-Solving

Problem-solving ability is one of the most important attributes for nurse managers to promote team integration to achieve maximum efficiency. Furukawa and Cunha (8) argue that, in nursing, problem-solving within teamwork emerged in the 1950s in the USA through experience and a solution to the issue of better use of personnel, as leaders develop and learn new skills and they demonstrate and use these skills in practice while setting teamwork as well as teaching others (9, 22, 24).

According to Aiken et al. (7), nursing leadership and problem-solving between groups increased significantly following an intervention and communication. Nurses' daily responsibilities are demonstrated by a critical path, a clinical path, or a care path that is an example of how problem-solving is weaved. To improve clinical problem-solving performance then, it would seem fruitful that nurses should be encouraged to develop a strong nursing leadership and well-structured knowledge base in the context of their discipline.

Hospitals do not provide education regarding problem-solving; thus, nurse managers shall have their own ability to solve a problem. Moreover, the nursing department or unit may develop its own module for nurses. Thus, this study postulates the following proposition to test whether problem-solving has a positive effect on nursing leadership:

**Hypothesis 4:** Problem-solving ability is positively related to nursing leadership.

### Transformational Role

One of the main roles of a nurse manager is to motivate followers and value specified and idealized goals, which are determined by the transformational role. A number of studies used the Leadership Practice Inventory approach to measure nurse managers in perception of leadership abilities to deemphasize that extraordinary nursing leadership composes of transformational roles. Using the method, Krugman and Smith (32) compared outcomes between two units: one with transformational leadership and the other one with conventional management. Their finding shows that nurses with transformational roles have a high rate to be nurse leaders, respected within an institution by departments and physicians.

Registered Nurses' Association (33) reports that support from colleagues with transformational qualities is important for nurse leaders. A transformational leadership ability of individuals broadens and motivates both parties to achieve greater levels of achievement, thereby transforming the work environment; moreover, it could be a great way to generate an optimum decision.

Highly and moderately relevant transformational roles are common among experienced nurses, while low and moderately relevant interpretations were more evident among young or non -experienced nurses (25). Researchers found that the transformational role of nurse managers is positively related to empowerment, and transformational leaders have a clear vision for the future and values in an ongoing dialogue with nurses. Nurse managers empower subordinates by motivating them to share in the vision and make it a reality; thus, they should have a transformational role to some extent. Consequently, the following hypothesis is set to examine whether the transformational role has a positive impact on nursing leadership:

**Hypothesis 5:** The transformational role is positively related to nursing leadership.

### Nursing Leadership

It is evident that leadership in nursing is of supreme importance at this time. The managerial career and nursing leadership are frequently seen as an award, an acknowledgment of a nurse's contribution to an organization and patient care services (8). Casida (6) discusses that a competitive leadership role is crucial for patient satisfaction and must be the survival of any healthcare facility that remains a priority of nurse managers. Nurse managers find themselves facing a challenging global nursing shortage—that the need for health care grows rapidly worldwide.

There are a variety of standards applicable to the practice of nursing leadership. The standards are based on the values of the profession, work environment, nursing actions, and interventions that a nurse implements to achieve desired outcomes in a particular hospital setting. Despite it, the hospital size is considered to be a fundamental feature with important implications for nursing leadership in hospital settings. Furthermore, nursing leadership is higher in bigger hospitals than in small ones (10).

Generally, it is acknowledged that one learns to be a leader by serving as a leader. One is a leader when he or she exercises leadership. Nurses progress throughout their careers as they face new challenges and conflicts in the workplaces. The establishment of criteria for the selection of nurse managers depends not only on years of experience but also on personality and management skills (4, 9, 12).

Nurse managers with positive leadership effects have their own self-interest for a higher purpose and stimulate followers, while those with negative leadership effects avoid leadership responsibilities and take action when issues become serious. When positive nursing leadership exists within nurse managers, patient satisfaction tends to be high, while turnover of nurse staff becomes low. Nevertheless, leadership policy shall be formulated by the human resources department, involving all management levels.

## Methods

To solve the problem of the sustainability of nursing leadership, the purpose of this study was to examine the relationship between nursing leadership and contributing factors to it, such as work environment, performance, behavior, problem-solving, and transformational role. We used a multifactor questionnaire survey method to collect data. This study has a descriptive and predictive design. Thus, the empirical data examination procedure consists of descriptive statistics, correlation, and multiple linear regression analysis.

### Sample and Design

During the study period, a total of 9,916 nurses worked in 2,881 health care settings of Mongolia, of whom 406 were registered nurse managers having worked as nurse managers for at least 1 year (17). On average, a nurse manager supervises 24 nurses. To design the sample, the first step consisted of listing all level health care institutions in Mongolia. These comprised 492 primary-level health care institutions, 28 secondary-level health care institutions, 21 tertiary-level health care institutions, and 2,340 other health care institutions, representing 128, 83, 79, and 116 nurse managers, respectively. Since the target population, 406, is not large, we purposively distributed the coded questionnaire to all nurse managers.

Questionnaires were distributed to the nurse managers of each participating hospital. The response rate achieved in this study was relatively high. All analyses were conducted at the 0.05 significance level. The participants were informed that the findings of this study may not benefit them directly, but, by being part of this study, they contribute to a better understanding of nurse leadership, patient care, and hospital structure of the Mongolian healthcare system. A copy of the summary of findings from the study was submitted to the Ministry of Health of Mongolia for a further policy implication. The coded questionnaire was taken from 205 nurse managers as over 50.4% of the total nurse managers in Mongolia in various hospitals of Ulaanbaatar and provinces. SPSS version 19 was used in data analysis.

The following procedures were employed to study the relationship between the dependent variable, nursing leadership, and the independent variables, including work environment, performance, behavior, problem-solving, and the transformational role. In each hospital, the head of nursing distributed the questionnaires to their nurse managers, and, when completed, they were collected from the nursing unit. The questionnaires were given to their home to respond with their convenience and returned a week later through the head or director nurses.

The response rate achieved in this study was comparatively good in comparison with other studies on nurse managers and leaders. Data collection that started in June 2013 was completed by September 2013. Basic demographic information about gender, age, education level, position level, and years of experience was added to the survey tool for all the participants to investigate how the demographics affect nursing leadership.

Study permission was obtained from seven hospital directors. All the participants had signed on the consent form prior to data collection and their rights to privacy and confidentiality.

### Instrument

The following are the seven parts of the survey questionnaire (see Appendix A).

1. Demographics include gender, age, education, position, and years of experience.
2. Fundamental features include organizational structure, basic knowledge of “leadership” and policy of particular hospital settings.
3. Work environment represents how nurse manager environment allows making autonomous nursing care decisions to suit patient needs that impact nursing leadership.
4. Performance represents how a nurse manager assesses nurse performance, how to decide to provide training sessions to teach new nursing technologies, develop new medical techniques, improve performance, anticipate and prevent misunderstanding/conflicts, redefine goals, consolidate teamwork for effective nurse leadership.
5. Behavior—how nurse managers enact the behaviors that convey support to staff and impact nursing leadership.
6. Problem-solving—how nurse managers effectively solve problems to be able to decrease the cost of health care and to increase the quality of patient care, and
7. Transformational role—how nurse managers adapt innovativeness of their approaches to the work and impact nursing leadership.

The five factors significantly contribute to nursing leadership that tested for the build, convergent, and distinguishable validity. The questionnaire consisted of a series of items with a five-point Likert scale (5 = strongly agree,…, 1 = strongly disagree) that reflects five factors of nursing leadership.

Fundamental features include the level of hospital size as to whether primary, secondary, tertiary, or other types of healthcare institutions. A few questions were asked from the participants to know nurses' knowledge about leadership and how hospital policy influences career development and nurse leadership. These fundamental questions are to identify an area of focus of nurses, hospitals, and to determine an area that needs attention to strengthen the effectiveness of leadership in the future.

### Operational Definitions

- Behavior of leadership is the ability to think critically, ability to solve problems, have respect for people, communicate skillfully, have the tendency to set goals, share vision, and have development of self and others (9).
- A healthcare institution is any hospital, convalescent hospital, health maintenance organization, health clinic, nursing home, extended care facility, or other institution devoted to the care of a sick, infirm, or aged person (18).
- Leadership is the position or function that organizes and guides a group of people to achieve a common goal and may or may not have any formal authority. The leadership role is building tolerance for ambiguity, setting performance standards for confidence, and holding subordinates accountable to those standards (18).
- Nurse is the protection, promotion, and optimization of health and abilities; prevention of illness and injury alleviation of suffering through the diagnosis and treatment of human responses and advocacy in health care for individuals, families, communities, and populations (33).
- A nurse manager is the nurse with management responsibilities of a nursing unit and requires strong leadership ability, clinical nursing knowledge, and decision–making within organizations employing nurses. The nurse manager does planning, organizing, staffing, directing, and controlling. The nurse manager is a middle manager who has 24-h responsibility for one or more hospitals or clinic units, regardless of the title assigned to that position. This position includes direct supervision of charge and staff nurses on all shifts and accountability for those positions [(4)].
- Performance is the accomplishment of a given task measured against preset known standards of accuracy, completeness, cost, and speed, which is the process of creating a work environment to enable perform best of the nurses' abilities [(25)].
- A problem solver is able to do direct and indirect interventions, delegation, purposeful inaction, consultation, and collaboration with others (4).
- A transformational role is “Four I's” as an idealized influence, inspirational motivation, intellectual stimulation, and individualized consideration (14).
- Work environment includes the surroundings, and conditions of influences that affected performance, role enhancement, and professional relationship in the short and long terms (21).

## Data Analysis

This section presents the demographics, analysis on fundamental features, correlation analysis, and multiple linear regression analysis.

### Demographics

This part is about participant demographics. Demographics include gender, age, education, work experience, and position with effects on both nurse retention and nursing leadership. First, descriptive statistics are used to describe the demographics of nurse managers.

Table 2 shows that 96% of the participants were female and only 9 male nurse managers. The data mean that nursing positions are dominated by the female group, which influences the leadership position as stated gender plays an important role in the nursing profession and remained predominantly female.

**Table 2:** Gender of participants.

		**Frequency**	**%**	**Valid %**	**Cumulative %**
Valid	Male	9	4.4	4.4	4.4
	Female	196	95.6	95.6	100.0
	Total	205	100.0	100.0	

Table 3 shows that 42% of the sample was aged 41–50 years old, 37.6% of them were 31–40 years old, 11.3% of the participants were 20–30 years old, and 9.3% of them were aged 51–60 years old, respectively.

**Table 3:** Age of the participants.

		**Frequency**	**%**	**Valid %**	**Cumulative %**
Valid	20–30 years	23	11.2	11.2	11.2
	31–40 years	77	37.6	37.6	48.8
	41–50 years	86	42.0	42.0	90.7
	51–60 years	19	9.3	9.3	100.0
	Total	205	100.0	100.0	

These data show that the majority of nurse managers aged between 31 and 50, which were able to gain work experience, the transition of knowledge, and clinical “know-how” from one generation of nurses to another, are imperative for nurse managers. Nurses with <1 year in the profession are more likely to quit their jobs. Nursing leadership makes older nurses stay in the workforce as long as they want by making a simple adjustment to the work environment.

Table 4 shows that 59.5% of the respondents have an associate diploma education, 40% of participants have a bachelor's degree, and only one nurse has a master's degree. All the nurses were graduated in Mongolia.

**Table 4:** Education of participants.

		**Frequency**	**%**	**Valid %**	**Cumulative %**
Valid	Associate Diploma	122	59.5	59.5	59.5
	Bachelor	82	40.0	40.0	99.5
	Masters	1	0.5	0.5	100.0
	Total	205	100.0	100.0	

Nursing education and the profession have a paralleled opportunity in today's health care system. Unfortunately, the current nursing education is not adequate to meet the needs of the future. Education must develop new partnerships with the community and healthcare institutions. More emphasis and resources must be directed to preparing bachelor's- and master's-level nurses that effective nursing leadership is grounded in the education of nurses in order to achieve successful outcomes.

Table 5 shows that 57% of the nurses are head nurses, 26% of them are registered nurses, about 10% of them are methodologist nurses, and around 6% of the participants are chief nurses.

**Table 5:** Position of participants.

		**Frequency**	**%**	**Valid %**	**Cumulative %**
Valid	Head nurse	118	57.6	57.6	57.6
	Nurse	54	26.3	26.3	83.9
	Director nurse	12	5.9	5.9	89.8
	Methodologist nurse	21	10.2	10.2	100.0
	Total	205	100.0	100.0	

Leadership myth is associated with the position. Moreover, the values of leadership involve occupying the top position in a hierarchy. A nurse manager is general terminology and divided into several positions, such as a director nurse of the nurse department; head nurses are senior nurses in a nurse unit or nurse department, and methodologist nurses are trainers of nurse staffs, who are supervised by the director of hospital settings, respectively. Nurses are former nurse managers; however, they currently hold the position of a nurse.

Table 6 shows that approximately 43% of the participants have 21–30 years of work experience, around 33% of them have 11–20 years of work experience, 16.6% of them have 0–10 years of work experience, and 7.8% of them have 31–40 years of work experiences. Data support the relationship between characteristics of the nurse manager workforce and the nurse leadership, which means nurses with longer work experiences are significantly more satisfied than their less-experienced colleagues with most of the facets of their work (34).

**Table 6:** Years of experience of the participants.

		**Frequency**	**%**	**Valid %**	**Cumulative %**
Valid	0–10 years	34	16.6	16.6	16.6
	11–20 years	67	32.7	32.7	49.3
	21–30 years	88	42.9	42.9	92.2
	31–40 years	16	7.8	7.8	100.0
	Total	205	100.0	100.0	

Our data show that between 11 and 30 years of work experience affects nurse managers' positions. Nurses with <10 years of work experience or more than 30 years of work experience do not hold a nurse manager position.

### Analysis of Fundamental Features

The study took place at public and private hospitals in the capital city, Ulaanbaatar, and other provinces of Mongolia. The nursing population was diverse, including large hospitals and small healthcare settings. Fifty-one nurse managers are from primary-level hospitals as 24.9% of total participants, 72 nurse managers are from secondary-level hospitals as 35.1% of the total participants and 40 nurse managers are from tertiary hospitals as 19.5% of the total participants, and 42 nurse managers are from other healthcare institutions as 20.5% of the total participants.

The primary hospitals require having 4–20 nurse staff, and one head nurse supervises other nurses, but it does not have a director nurse or a methodologist nurse. Every secondary and tertiary hospital must have a nurse department consisting of one nurse director, two to five methodologist nurses, and around 20 heads in order to manage 250 nurse staff. Other hospital settings, such as healthcare departments in 21 provinces and Ulaanbaatar city, must have at least one nurse manager, either in the position of a director nurse or head nurse.

In addition, we investigated whether nurse managers have knowledge about “leadership”; hence, first questions were “Do you know the word “Leadership?” About 157 nurse managers or 76.58% of the total participants know about it; unfortunately, 48 nurse managers or 23.41% of the total participants do not know about the term “leadership.”

Also, some policy-related questions were asked and analyzed as follows. First, “Do hospital policies and procedures have to support the leadership of nurse managers?” About 118 nurse managers or 57.5% of the total participants answered “Yes,” 38 or 18.5% answered “No,” and 49 nurse managers or 23.9% gave an answer of “Do not know.” Second, “Does a nurse manager influence mission and decision-making of general administration issues of the organization?” About 161 nurse managers or 78% of the total participants answered “Yes,” 33 or 16% answered “No,” and 11 nurse managers or 5.3% gave an answer of “Do not know.” Third, “What level of leadership responsibility does nurse manager need?” About 161 nurse managers or 78.5% said “High,” 33 nurse managers or 16% said “Medium,” and 11 nurse managers or 5.5% of the total participants said “Low.”

These fundamental questions are considered to know nurse managers' complaints and suggestions about leadership in the nursing department and hospital settings. Managers who talk to their staff on a regular basis are more informed and have less difficulty when situations occur and increase job satisfaction of nurses, furthermore effects to nursing leadership. Nurses should participate in the policy arena and the decision-making procedure and be engaged in health care reform-related implementation efforts. Increasing the involvement of nurses in high-level leadership contributes to a more stable workforce and, in turn, positively impacts patient quality and safety and transparency and accountability of hospital settings structure.

### Correlation Analysis

The relationship between the dependent variable as nursing leadership and five independent variables as work environment, performance, behavior, problem-solving, and transformational role was examined using correlation analysis. Significance was tested at the alpha = 0.05 level. Correlation studies are appropriate when there is a need to clarify the relationship, and little or no previous research has been undertaken. Possible relationships were examined using Pearson correlation coefficients shown in Table 7.

**Table 7:** Pearson correlation.

		**WE Ave**	**P Ave**	**B Ave**	**PS Ave**	**TR Ave**	**NL Ave**
WE Ave	Pearson Correlation	1					
	Sig. (2-tailed)						
	N	205					
P Ave	Pearson Correlation	0.143	1				
	Sig. (2-tailed)	0.040					
	N	205	205				
B Ave	Pearson Correlation	0.052	0.121	1			
	Sig. (2-tailed)	0.458	0.084				
	N	205	205	205			
PS Ave	Pearson Correlation	0.030	0.261	0.269	1		
	Sig. (2-tailed)	0.672	0.000	0.000			
	N	205	205	205	205		
TR Ave	Pearson Correlation	−0.015	0.185	0.143	0.159	1	
	Sig. (2-tailed)	0.836	0.008	0.041	0.022		
	N	205	205	205	205	205	
NL Ave	Pearson Correlation	0.047	0.092	0.904	0.367	0.159	1
	Sig. (2-tailed)	0.505	0.189	0.000	0.000	0.023	
	N	205	205	205	205	205	205

*WE, work environment; P, performance; B, behavior; PS, problem-solving; TR, transformational role; NL, nurse leadership*.

In terms of the independent study variables, two were found to have a significant effect on nursing leadership: behavior and performance of nurses significantly (*p* < 0.05) to nursing leadership positively. The transformational role moderately (*p* < 0.05) intercorrelated with nursing leadership. However, the work environment and performance were found not to be strongly related to nursing leadership when entered with the other independent variables.

Table 7 shows the results of a Pearson correlation coefficients; nurse leadership (*n* = 205), informed that there was a strong correlation r (205) = 0.90, *p* = 0.000 between the behavior and nurse leadership and r = 0.36, *p* = 0.000 between problem-solving and nurse leadership. Also, the transformational role and nurse leadership produced a positive correlation r = 0.159, *p* = 0.023. However, there is no relationship between performance and nurse leadership r = 0.092, *p* = 0.189, and between work environment and nurse leadership r = 0.047, *p* = 0.505.

The results suggested that successful nurse leadership is based on behavior and problem-solving. This opens the floodgates to nurse leadership development, as opposed to simple psychometric assessment that sorts those with leadership potential from those who will never have the chance. Leaders must be taught how to adapt and change constantly to keep up. Also, problem-solving is the most crucial and common thinking process used in nursing that requires various mind actions. This enables them to more accurately represent the nature of the clinical problem and to deal with the problem less in sequential terms in order to override clinical concepts. Thus, the findings support Hypotheses 3 and 4.

The majority of nurse managers are female, and the female leaders scored higher than the male leaders on all transformational roles, because it provides them with a means of overcoming the dilemma of the role and ability to meet the requirement of their leadership role. Therefore, this study supports Hypothesis 5 that the transformational role positively affects nursing leadership.

The work environment and performance are outcome variables that are determined to be mediated by the workload of nurses (3); however, the findings of this study do not support Hypotheses 1 and 2 that variables significantly low contributes to nursing leadership at the hospital level. These results show that, in Mongolia, nursing leadership is strongly correlated with behavior, problem-solving, and transformational roles, and nurses' performances and work environment must be improved to create a professional practice environment for nurse managers.

### Multiple Linear Regression Analysis

Inferential statistics, including R-square, regression, and multiple linear regression analysis, are used to test the validity of the set hypotheses. Multiple linear regression analysis determines whether nurse leadership perceives work environment, performance, behavior, problem-solving, and transformational role. The linear combination of the five independent variables was significantly related to the dependent variable (nurse leadership), R squared = 0.83, adjusted R squared = 0.83, or 83% of the total variance in the dependent variable.

Table 8 contains the ANOVA and shows the factors that contribute to nursing leadership. The analysis shows that there is a difference with an F score of 5, 199 = 204.81 and significance (0.000) well-beyond the alpha < 0.05 standard.

**Table 8:** Multiple linear regressions for a single set of predictors: a model summary.

**Model**	**R**	**R Square**	**Adjusted R Square**	**Std. Error of the Estimate**	
**Model Summary**					
1	0.915	0.837	0.833		0.3007438
**Sum of squares**		**df**	**Mean square**	**F**	**Sig**.
**ANOVAb**					
Regression	92.622	5	18.524	204.810	0.000
Residual	17.999	199	0.090		
Total	110.621	204			

The multiple linear regression analyses showed that behavior and problem-solving positively contribute to nursing leadership. But work environment, performance, and transformational roles do not contribute to nursing leadership. The level of statistical significance was set *a priori* at = 0.05. Table 9 shows that the model analysis included the five independent variables of the work environment, performance, behavior, problem-solving and transformational ability. The behavior (*t* = 29.058, *p* < 0.05) and problem-solving (*t* = 4.693, *p* < 0.05) are emerged as a significant coefficient of the dependent variable. No other variables in the model were significant.

**Table 9:** Multiple linear regressions for a single set of predictors: coefficients.

	**Unstandardized coefficients**		**Standardized coefficients**		
	**B**	**Std. Error**	**Beta**	**t**	**Sig**.
**Coefficients**					
(Constant)	−0.015	0.219		−0.067	0.947
Work environment	0.006	0.031	0.006	0.192	0.848
Performance	−0.104	0.057	−0.056	−1.836	0.068
Behavior	1.017	0.035	0.869	29.058	0.000
Problem-solving	0.155	0.033	0.144	4.693	0.000
Transformational role	0.025	0.033	0.022	0.750	0.454

There is, therefore, a need to develop a work environment in a hospital setting and enhance performance and encourage transformational roles in order to strengthen the effectiveness of nursing leadership.

The results from the regression equation for the standardized variables were as follows: Predicted work environment score = 0.006 + (−0.104) (performance) + 1.017 (behavior) + 0.155 (problem solvency) + 0.025 (transformational ability) (Table 9). The findings provide support for the hypotheses (H3 and H4). These findings answer Research Questions 1 and 2 positively. The 0.000 significance level is less than the level of significance for the test of (0.05). However, the findings do not support Hypotheses 1 and 2, and weak support Hypothesis 5. Behavior was determined to be the strongest predictor of the five variables, and work environment was the weakest predictor of nursing leadership.

Nurse managers must have positive behavior and capable problem-solvers because their profession requires a high level of cognitive reasoning and discretionary decision-making that supports Hypotheses 3 and 4 as behavior and problem-solving contribute to nursing leadership. The transformational role is more focused on processes that motivate followers to perform to their full potential by influencing change and providing a sense of direction for nurse managers. Therefore, this study found that the transformational role slightly contributes to the nursing leadership as finding supports Hypothesis 5.

Minimizing nurse staff workload and enhancing nurse staff job satisfaction should be consistent with retaining nurse leaders in the profession. Unfortunately, this study does not support Hypothesis 1 that the work environment does not contribute to nursing leadership. The nurse manager must assess and improve nurse staff's performance, decide to provide training sessions to teach nursing technologies, and consolidate teamwork. But this study does not support Hypothesis 2 that performance does not contribute to nursing leadership.

## Conclusions and Discussions

To solve the problem of the sustainability of nursing leadership, the purpose of this study is to examine the factors that contribute to nurse leadership in hospital settings in Mongolia. This section discusses the findings in relation to the theoretical framework, stated limitations, and presented suggestions and concluding remarks on the further implication of research.

This study is the first research in the literature to assess nursing leadership in Mongolia. Correlation coefficients give the direction of causation in the relationships of variables. According to the results of multiple linear regression analysis, two of the variables, namely, behavior and problem-solving, have strong positive influence on nursing leadership. Nonetheless, work environment and the transformational role do not have significant impact on nursing leadership. Finally, performance has a weak significant influence on nursing leadership.

This study is essential to develop nursing practice, increase the reputation of nurses, and motivate nurses to work in hospital settings for independent decision-making of patient care. Leadership is an observable, learnable set of practices with the desire and persistence to lead—to make difference—that can substantially improve nurse abilities.

The realities of a global society, expanding technologies, and an increasingly diverse population require nurses to master complex information, to coordinate a variety of care experiences, to use technology for health care delivery and evaluation of nursing outcomes, and to assist clients with managing an increasingly complex system of care, which wholly requires to have nursing leadership.

Nursing leadership promotes harmonious interaction between persons and their environment, strengthens the wholeness of an individual, and redirects human and environmental patterns or organization to achieve maximum health. The nursing leadership congress is designed to help nurses become catalysts, and it provides an opportunity to share practical experiences in solving many problems in the health care industry. It focuses mainly on practical experience rather than a theoretical approach. By postulating new factors and relationships and confirming the relevance of leadership factors and their relationships, the study has opened up new horizons for other researchers to investigate more deeply and precisely.

### Discussion

Whereas correlation coefficients give the direction of causation in the relationships of variables, the multiple linear regression analysis attempts to explore the relationship between independent and dependent variables. Hypothesis tests were performed to answer the following research question as “How do specific factors (work environment, performance, behavior, problem-solving and transformational role) contribute to nursing leadership in Mongolia?”

The finding of this study says that two of the variables, namely, behavior and problem-solving, positively contribute to nursing leadership and nurses' perceptions of their leader's effectiveness. This means that this study supports two out of the five hypotheses and does not support three hypotheses. The results suggest that an individual behavior and characteristics (problem-solving ability and the transformational role) strongly reflect leadership. In contrast, the reflection of external variables depends on the profession and specialty, as nurses have a high workload; therefore, work environment and performance do not contribute to the nursing leadership.

The nurse department consists of nurses with different types of behaviors, but individual behavior affects the outcome of the nurse leadership. Registered Nurses' Association (33)'s guideline states that the individual behavior of a leader is important, but, also, the culture, climate, and values of organizations are essential to building the behavior of an individual. Since nursing research is not common in Mongolia, it is necessary to explore the way how behavior influences nursing leadership and, in turn, how the behavior of the nurse leadership influences the organizational outcome.

#### What Are the Multiple Correlations Between the Predictors (Work Environment, Performance, Behavior, Problem-Solving, and Transformational Ability) and the Nursing Leadership?

The multiple regression performed in this study indicated 83% of the variance in nursing leadership was accounted for by the linear combinations of work environment, performance, behavior, problem-solving, and the transformational role. Therefore, it is important to explore variable factors to impact nursing leadership in hospital settings in Mongolia.

The results in this study revealed a positive correlation existed between the dependent variable, nursing leadership, and three independent variables, behavior, problem-solving, and the transformational role. Behavior reflected the strongest correlation, followed by perceived problem-solving and the transformational role of nursing leadership. This means that nurse leaders should accurately anticipate and prevent misunderstanding and conflicts, redefine the goals of nurse managers, develop new medical techniques, and facilitate desirable strategic decision-making.

Registered Nurses' Association (33) identified that there is a growing understanding of the relationship between nurses' work environment, patients' outcomes, and healthcare institutions' performances. However, our study did not confirm that the work environment influences nurse leadership. Moreover, there is some research on the direct impact of the work environment on developing and sustaining nursing leadership. Nurse manager turnover is usually associated with a range of negative outcomes, including training new nurses, increased workload, and the salary range.

This research suggests that gender roles are higher from their management identity as nurse managers in hospital settings. For those who evaluate the competence and effectiveness of nursing leadership in hospital settings that are mostly female, the data suggest that females may be more effective leaders since females are more likely to practice a transformational role. This is a very important implication in order to develop a policy framework for health care settings.

#### Why Do We Need to Study Factors Contributing to the Sustainability of Nursing Leadership?

When we know the factors that contribute to nursing leadership, healthcare institutions are able to develop leadership styles among nurses in the nursing department. The study increases the effectiveness of current nurse managers and guides the identification of future nurse leaders.

Currently, in Mongolian hospitals, almost more than 50 percent of nurse managers' performances spent for administrative work include making a list of all the prescription drugs, counting the number of beds and linens in a hospital, and monitoring shift change of nurse staffs. Therefore, very few percentages of performances are spent on hospital care. Thus, performance was not contributed to the nurse leader in the Mongolian case. In the future, it must change the nurse manager's role that enables high performance for quality care of patients and hospital care.

Moreover, nurse managers experience a higher workload than ever before due to several reasons, although the work environment does not support nurse leadership. The reasons are, first, hospitals do not have online patient registration; therefore, the nurse managers fill out all registration forms by hand, and, hence, they spend most of their working hours in the workplace. Second, there is no consolidated database of nurse performance within hospitals, compared to the physicians. For instance, hospitals have an integrated database for all physicians; however, neither nurse managers nor nurses have an integrated database. Third, the high workload of nurse managers does not allow training other nurse staff due to shortage of time. Finally, there is a lack of technology, including the internet environment and patient care resources.

The specialty nursing expertise is generally obtained on the job, also through nursing programs to attract new graduate nurses and motivate them further in nursing leadership. In Mongolia, around 1,000 nurses graduate from the National Medical University and its three branches, and private three universities per year. Nursing leadership programs must be offered through undergraduate and graduate education in formal and informal ways. Unfortunately, currently, nursing leadership programs are offered neither by universities nor hospitals. High school graduates are less likely to major by the nurse due to low reputation and low career development. Moreover, promoting higher education to nurses of all educational levels is critical to developing nurse leadership in hospital settings. Hence, another main reason that why the work environment and performance of nurse managers do not support our hypotheses.

#### How Does Nurses' Role Function Transfer to a Leadership Role in the Hospital Care Delivery System?

Nurse managers' autonomy over decisions affects the work at the unit level, patient care services, and health care institutions' commitment. When nurse leadership is high among nurses, nurse managers feel empowered and influential not only in their current role but also regarding impacts on nursing staff.

Leadership is rewarding and important for building succession, and it is a significant level of commitment to a job (18). But, in the Mongolian case, it is controversial as nurses are at the same level as kindergarten teachers and elementary school teachers; unfortunately, their salary range is lower than theirs. A nurse manager earns only one percent higher salary than nurses; however, less-experienced nurse managers have the same salary range as nurses. Therefore, the performances of nurse managers that are weak, do not motivate them to be leaders. In the last few years, the education level of nurse managers has been increased, and almost 50 percent of nurses have a bachelor's degree. However, the higher education level does not increase salary.

Promotion is not common among nurse managers and nurses that raise a negative impact to nurse performance and nurse leadership. The performance assessment is not clear in hospital settings. The nurse service quality is far away from the international standards; therefore, patients have more complaints on nurse performance, which directly affects nursing leadership. Quality of care is based on confidence and competence, which nurse leaders need support now more than ever.

Physicians and doctors do not recognize the nurse leadership role in patient care service and do not have the legal environment to support the nurse manager's performance and work environment.

#### Is Nursing Leadership Essential for a Hospital? If Yes, How?

The nurse staff is working longer hours and taking an increased patient assignment. Moreover, job satisfaction highly reflects nurse turnover. Therefore, involving nurse staff at a high level in policy and procedure development will score high on the retention scale and motivate nurse leadership among nurse managers in hospital settings. Moreover, strengthening nursing leadership is particularly critical not only in nursing and medicine but also in society.

Head nurses and director nurses include the members of management of a hospital; indeed, they must be involved in decision-making for patient care and policy development of an organizational structure. Unfortunately, nurse managers have a weak nurse leadership role, which cannot reflect strong policy development in a whole organizational setting.

Overall, these results suggest an important role of nurse leadership in strengthening hospital development and patient care services in hospital settings. The nursing unit must set behavioral standards, problem-solving approaches, and transformational roles among nurses that most positively influence the nursing leadership. On the other hand, the external variables, as work environment and performance, have to reflect the demanding role of today's nurse managers at the surface level of a hospital.

### Limitations

The data were gathered using a self-report questionnaire, like the majority of earlier studies, and no objective measures were used. Self-report data might be contaminated by common method variance because five independent variables and dependent variables are based upon one source of information. Nevertheless, this study has stated that leadership has a strong and positive impact regardless of whether outcomes are measured subjectively or objectively.

Future studies need to identify the work environment and performance of nurses in the hospital settings in regard to nursing populations.

### Suggestions

Health care organizations must invest in educational programs to develop leadership competencies in the workplaces to enhance their roles. Accordingly, the Ministry of Health of Mongolia must organize fruitful leadership programs in that nurses and new graduates should be encouraged to develop a strong and well-structured knowledge base in the context of their discipline. The curriculum should make an explicit reference to the international experience base and further development of nurses.

Nurse managers must have the higher professional expertise to sustain nursing leadership comparing nurse staff; however, there are no criteria between nurse managers and nurse staff to compare the effectiveness of leadership roles. An online database of nurses and nurse managers must be developed, and promotional activities are vital for effective nursing leadership.

Recently, the educational level of nurses has been increased, but there are no differences in terms of reference between position levels of nurses. The key recommendation is for the reinvention of nursing education and work environments to address and appeal to the needs and values of a new generation of nurses and enhance the quality of patient care.

Effective nursing practice, education, research, and leadership are grounded in the complexity of human relationships and, therefore, require systematic and careful thinking in order to achieve successful outcomes of nurse performance. A hospital organizational structure must allow having a voice in policymaking for nursing service and patient care. We need a stronger model for developing and grooming nurse leaders. The nurse career model must include differential salary ranges between nurses and nurse managers that positively impact nursing leadership.

Currently, the basic techniques in hospitals are very old, and they must change the techniques in a complex way and renovate the hospital buildings, which can impact the work environment and enhance patient care services. Unfortunately, due to financial shortage, Government is not able to support hospitals, which has negative reflects on nursing leadership. The supply of hospital equipment and linens is not sufficient for hospital settings; therefore, we have widely recognized the quality of supply apart from product quality that strengths the work environment of nurses and, moreover, impacts the nursing leadership.

The policy of hospitals has greater uncertainty and ambiguity; therefore, in the forthcoming years, we will likely see greater revision and practical approaches to promote nursing leadership. Moreover, it is necessary to collectively determine the purpose of nursing leadership and to make changes in our healthcare systems that positively impact patient care services. This guiding purpose will help us determine what we are likely to do, and where we are likely to go from here. Our paper applies descriptive and correlation analysis and employs multiple linear regression models to examine nurse management and patient care services. Extensions of our paper include using our approach to examine food waste reduction (35, 36), network analysis (37), carbon emissions (38), procurement system (39), and many others. Readers may read Wong (40) for other areas in that academics and practitioners could apply the approach used in our paper for their studies. This paper studies the sustainability of nursing leadership; scholars can apply the approach used in this paper to study the sustainability of herding behavior ((41)), portfolio selection (42), organizational climate and work style (43), supply chains (44), health insurance (45), and many others.

## Data Availability Statement

The raw data supporting the conclusions of this article will be made available by the authors, without undue reservation.

## Ethics Statement

Ethical review and approval was not required for this study in accordance with the local legislation and institutional requirements. The participants provided their written informed consent to participate in this study.

## Author Contributions

BW, DD, and M-UB: conceptualization. BW: methodology. DD: data curation and writing—original draft. BW and DD: formal analysis. W-KW: supervision. M-UB and OS: writing—review and editing and funding acquisition. W-KW and M-UB: project administration. All authors contributed to the article and approved the submitted version.

## Funding

This research was supported by Chinese Academy of Medical Sciences and Peking Union Medical College, China (Grant number: 2021-RC630-001), National University of Mongolia, Asia University, China Medical University Hospital, The Hang Seng University of Hong Kong, Research Grants Council (RGC) of Hong Kong (project numbers 12502814 and 12500915), and the Ministry of Science and Technology (MOST, Project Numbers 106-2410-H-468-002 and 107-2410-H-468-002-MY3), Taiwan. However, any remaining errors are solely ours.

## Conflict of Interest

The authors declare that the research was conducted in the absence of any commercial or financial relationships that could be construed as a potential conflict of interest.

## Publisher's Note

All claims expressed in this article are solely those of the authors and do not necessarily represent those of their affiliated organizations, or those of the publisher, the editors and the reviewers. Any product that may be evaluated in this article, or claim that may be made by its manufacturer, is not guaranteed or endorsed by the publisher.
