# Supplementary material for: Sustainability of Nursing Leadership and Its Contributing Factors in a Developing Economy: A Study in Mongolia
Source: Front Public Health. 2022 May 25;10:900016. doi: 10.3389/fpubh.2022.900016 (PMC9174683; doi:10.3389/fpubh.2022.900016)
Supplement: Supplementary file 1 [file Data_Sheet_1.pdf]

Appendix A

	Variable	
1	Gender	[ ] Male
		[ ] Female
2	Age	[ ] 20-30 years
		[ ] 31-40 years
		[ ] 41-50 years
		[ ] 51-60 years
		[ ] 61-70 years
3	Education	[ ] Associate
		[ ] Diploma
		[ ] Bachelor
		[ ] Masters
		[ ] Other
4	Position	[ ] Head nurse
		[ ] Nurse
		[ ] Director nurse
		[ ] Methodologist nurse
		[ ] Other
5	Years Experience	[ ] 0-10 years
		[ ] 11-20 years
		[ ] 21-30 years
		[ ] 31-40 years
		[ ] 41-50 years
6	Years in Position	[ ] 0-5 years
		[ ] 6-10 years
		[ ] 11-15 years
		[ ] 16-20 years
		[ ] 21-25 years
		[ ] 26-30 years
		[ ] 31-35 years

ORGANIZATIONAL STRUCTURE		
1	What level is your hospital?	[ ] Primary
		[ ] Secondary
		[ ] Tertiary
2	Do you know the word “Leadership?	[ ] Yes
		[ ] No
3		[ ] Yes
		[ ] No

	Do hospital policies and procedures have support the leadership of nurse managers?	[ ] I do not know
4	What level of leadership responsibility does nurse manager?	[ ] High
		[ ] Medium
		[ ] Low

Please indicate the extent to which you agree or disagree with the following statements:
(1 – Strongly disagree, 2 – Disagree, 3 – Neutral, 4 –Agree, 5 - Strongly agree)

WORK ENVIRONMENT		
1	You have a stimulating, intellectual environment	1 2 3 4 5
2	The work associated with your position provides you with the opportunity to use a full range of nursing skills	1 2 3 4 5
3	The nursing manager environment allows you to make autonomous nursing care decisions to suit patient needs	1 2 3 4 5
4	Nurses receive adequate praise for work well done from nursing administration	1 2 3 4 5
5	The hospital organizational structure allows you to have a voice in policy making for nursing service	1 2 3 4 5
6	Nurse manager has an enough time to participate in patient care procedure	1 2 3 4 5
7	Good working relationships exist between you and your peers, including supervisor and physicians	1 2 3 4 5
8	Does your hospital have electronic registration for nursing care service	1 2 3 4 5
9	Encourages you to make adjustments in your nursing practice to suit patient needs	1 2 3 4 5
10	Your working environment offers an opportunity for professional growth	1 2 3 4 5
11	I make sure that my part in the group is understood by the group members	1 2 3 4 5
PERFORMANCE		
1	Position description of nurse manager is clear and adequate	1 2 3 4 5
2	**Performance of nurse's manager is directions of patient health care procedure**	1 2 3 4 5
3	**Nurse manager have nursing plan for each patient and check performance nursing procedure**	1 2 3 4 5
4	Nurse manager schedules the work to be done.	1 2 3 4 5
5	Nurse manager leads team nurses performance every day.	1 2 3 4 5
6	Nurse manager’s teaching ability is good	1 2 3 4 5
7	Hospital supply new medical nursing techniques by nurse manager’s intention	1 2 3 4 5
8	**Nurse managers care about professional development and medical research**	1 2 3 4 5
9	Nurse manager evaluates comments or complain issued by patient about nursing care	1 2 3 4 5
10	Monitoring and controlling the performance of nursing support staff within the unit.	1 2 3 4 5
11	Nurse manager asks that group members to follow standard rules and regulations.	1 2 3 4 5
BEHAVIOR		
1	I consider the moral and ethical consequences of decisions.	1 2 3 4 5
2	I am friendly and approachable	1 2 3 4 5
3	I overcome attempts made to challenge my leadership	1 2 3 4 5
4	I drive hard when here is a job to be done	1 2 3 4 5
PROBLEM SOLVER		
1	I can make accurate decisions.	1 2 3 4 5

2	Autonomous nurse manager has to make decision for patient care	1 2 3 4 5
3	The nursing department policy has to get nurse for problem-solving through direct way	1 2 3 4 5
TRANSFORMATIONAL ABILITY		
1	I go beyond self-interest for the good of the group.	1 2 3 4 5
2	I make clear what one can expect to receive when performance goals are achieved.	1 2 3 4 5
3	I permit the members to use their own judgment in solving problems	1 2 3 4 5
NURSE LEADERSHIP		
1	How much do you know about nursing leadership management?	1 2 3 4 5
2	What level do you think developing model nurse manager?	1 2 3 4 5
3	Do hospital policies and procedures have support the leadership of nurse managers?	1 2 3 4 5
